# Effect of Composition and Size on Surface Properties of Anti-Cancer Nanoparticles

**DOI:** 10.3390/ijms241713417

**Published:** 2023-08-30

**Authors:** Ina Mishra, Meredith Garrett, Stephen Curry, Jeffrey Jameson, Michail Kastellorizios

**Affiliations:** Department of Pharmaceutical Sciences, University of North Texas System College of Pharmacy, University of North Texas Health Science Center, Fort Worth, TX 76107, USA

**Keywords:** interfacial tension, liposomes, nanoparticles, clinical translation, Doxil^®^

## Abstract

Liposomal formulations offer significant advantages as anticancer drug carriers for targeted drug delivery; however, due to their complexity, clinical translation has been challenging. In addition, liposomal product manufacturing has been interrupted in the past, as was the case for Doxil^®^ (doxorubicin hydrochloride liposome injection). Here, interfacial tension (IFT) measurements were investigated as a potential physicochemical characterization tool to aid in liposomal product characterization during development and manufacturing. A pendant drop method using an optical tensiometer was used to measure the interfacial tension of various analogues of Doxil^®^ liposomal suspensions in air and in dodecane. The effect of liposome concentration, formulation (PEG and cholesterol content), presence of encapsulated drug, as well as average particle size was analyzed. It was observed that Doxil^®^ analog liposomes demonstrate surfactant-like behavior with a sigmoidal-shape interfacial tension vs. concentration curve. This behavior was heavily dependent on PEG content, with a complete loss of surfactant-like behavior when PEG was removed from the formulation. In addition to interfacial tension, three data analyses were identified as able to distinguish between formulations with variations in PEG, cholesterol, and particle size: (i) polar and non-polar contribution to interfacial tension, (ii) liposomal concentration at which the polar and non-polar components were equal, and (iii) rate of interfacial tension decay after droplet formation, which is indicative of how quickly liposomes migrate from the bulk of the solution to the surface. We demonstrate for the first time that interfacial tension can be used to detect certain liposomal formulation changes, such as PEG content, encapsulated drug presence, and size variability, and may make a useful addition to physicochemical characterization during development and manufacturing of liposomal products.

## 1. Introduction

Nanoparticles are a promising technology to improve cancer diagnosis and therapy compared to conventional treatment and diagnostic methods. Therefore, increasing efforts are being made to formulate and test anticancer nanoparticles and expand the application of such formulations. The nanomedicine field in biomedical and drug delivery applications is ever-expanding, with a strong emphasis on anticancer drug delivery [1,2], as well as a renewed emphasis on immunotherapy [3] and vaccine development [4].

Many formulations successful in preclinical stages enter clinical trials [5]. However, there has been limited clinical translation of nanoparticles and at present there are only eight FDA-approved nanomedicines for the treatment of cancer [5,6]. Nanoparticle formulations are fabricated with methods that are not easily adaptable to large-scale manufacturing [7,8,9]. The techniques inherently produce products with similar, yet not identical, ‘nano-properties’ such as size distribution, that lead to batch-to-batch variation. These variations cause reproducibility and regulatory issues that affect large-scale production [7,10,11]. Currently, no well-defined criteria exist for liposomal products, meaning for each product, regulatory studies must be conducted to determine and extensively evaluate critical quality attributes (CQAs) to establish appropriate ranges of each attribute for quality assurance [12,13]. Since the CQAs of each product may differ, this process significantly slows the progress of liposomal drug development. Liposome size as well as particle size distribution (PDI) are common CQAs, but more characteristics that affect physicochemical properties of liposomes are needed to better establish a quality control process. Here, we show that IFT is sensitive to changes in size, PDI, and formulation content and may serve as a potential method for quality assurance of liposomal formulations.

The importance of nanoparticle surface biochemistry as well as liposomal surface properties and their manipulations that affect liposome circulation in bloodstream are well known [14,15,16]. Interaction of conventional liposomes and serum components, such as albumin or neutrophils, trigger the primary immune response and lead to clearance of the nanoparticle [17,18]. Surface modification of conventional liposomes using hydrophilic polymers such as polyethylene glycol (PEG) was a milestone in anticancer nanomedicine [19,20]. PEG inhibits interaction with serum components and therefore helps the liposome evade the primary immune response [21,22]. These stealth liposomes have an increased survival and efficacy and may also decrease clinical adverse effects. However, more knowledge about surface properties and their interactions with the surrounding environment during transport is required to understand the intra-tumoral distribution of liposomes [23,24,25,26].

Surface properties such as interfacial tension and wettability influence a particle’s interaction with its surroundings [27,28]. The high curvature of nanoparticles increases their surface activity and can cause differences in the formation of the ‘protein corona’ [29,30]. However, further understanding of the contributions of surface properties such as interfacial tension to nanoparticle-protein interactions is needed.

Interfacial tension of liposomes has been suggested to affect aggregation and determine stability. Such studies in the past consisted of non-PEGylated multilaminar vesicles (MLVs) containing lipophilic drug; the lipid composition and size differed from the current clinical liposomes for cancer treatment such as Doxil^®^ [31]. Surface properties of PEGylated liposomes have not been studied. To our knowledge, this is the first study to investigate surface properties of PEGylated liposomes, controlled for size and homogeneity, and identify component(s) responsible for their surface properties. The present work represents part of an ongoing study in our laboratory to understand surface interactions of clinically used liposomes. In this paper, we quantified interfacial tension for different PEGylated liposomes and identified components crucial for the surface activity of these liposomes.

In this study, we used Doxil^®^ as the prototype to make in-house liposomes and understand their surface properties. Doxil^®^ is an FDA-approved anticancer nanoparticle formulation used against many types of cancer [20]. Doxil^®^ nanoparticles consist of doxorubicin (anticancer drug) encapsulated in the core of the liposome. Its lipid bilayer is made up of hydrogenated soy phosphatidylcholine (HSPC) and cholesterol with PEG molecules on its surface; lipid molar ratio 11:5:1 or 9.6, 3.2, 3.2 mg/mL. We studied the surface properties of in-house liposomes that mimicked the lipid composition of Doxil^®^ and varied composition and size to identify the component(s) contributing to liposomal surface activity.

This study lays down an important building block toward better understanding liposomal surface interaction before uptake by the cancer cell. The results from this study will be useful in understanding the optimum lipid ratio for cancer drug delivery and thereby improving clinical translation of liposomes.

## 2. Results and Discussion

The goal of this work was to investigate interfacial tension as a potential physicochemical characterization tool for liposomal products. The formulation of liposomal doxorubicin (Doxil^®^) was used as a reference, and formulations with variations in composition and particle size were analyzed at different concentrations using an optical tensiometer. The optical tensiometer measures interfacial tension by capturing a video of a droplet dispensed by a syringe and analyzing its shape.

### 2.1. Effect of Liposome Size on Interfacial Tension

Mean hydrodynamic diameters for all formulations are shown in Table 1. All formulations were monodispersed, with PDI values between 0.02 and 0.04, and average diameter similar to or double Doxil^®^, as appropriate.

**Figure 1:**
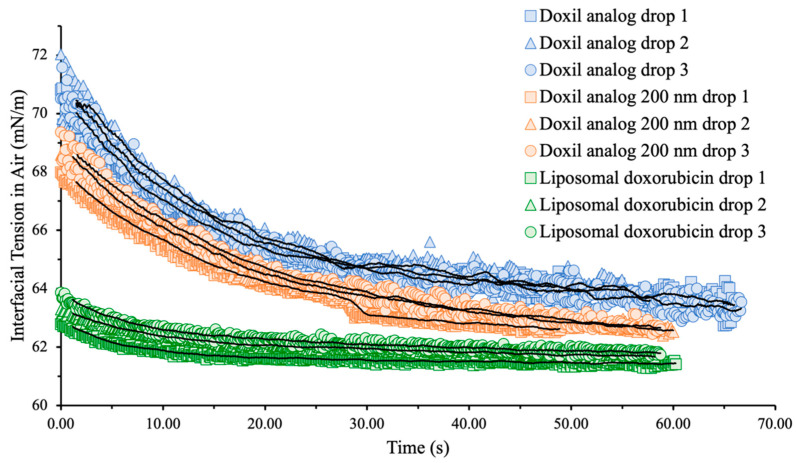
Interfacial tension (mN/m) vs. time (s) raw data obtained for Doxil^®^ analogs and liposomal doxorubicin at total lipid concentration 0.5 mg/mL. Solid black lines are moving averages of each sample.

To study the effect of liposome size on interfacial tension, liposomes with diameter similar to Doxil^®^ (100 nm) and double to Doxil^®^ (200 nm) were studied at 0.5 mg/mL with doxorubicin-loaded liposomes as a control. As shown in Figure 1 and Table 2, while interfacial tension values of Doxil^®^ analogues of different sizes are similar, the rates at which these values were reached are different. The rate by which the interfacial tension reaches a plateau is indicative of how quickly surface-active components migrate from the bulk of the liquid to the surface. Larger liposomes exhibited a slower migration to the liquid surface and resulted in a slightly lower interfacial tension than liposomes with diameter similar to Doxil^®^. Particle size is known to have an effect on how nanoparticles interact with biological interfaces across several applications, such as formation of a protein corona [32], uptake and cytoprotection in brain cells [33], blood–brain barrier penetration [34], and pharmacokinetics [35]. The observed effect of particle size on interfacial tension of liposomal suspensions reported here raises the question whether a correlation between interfacial tension and biological action exists and could potentially be used as a tool to fine-tune nanoparticle formulations in the preclinical stage.

Additionally, doxorubicin-loaded liposomes had a lower interfacial tension than empty analog formulations no matter the size and reached their plateau value significantly faster. This may be due to the drug affecting the vesicle’s density. Density difference can also explain the variation in interfacial tension decay between larger and smaller liposomes, since larger liposomes are significantly less dense than smaller ones and move more slowly.

### 2.2. Effect of Liposome Concentration on Interfacial Tension

**Figure 2:**
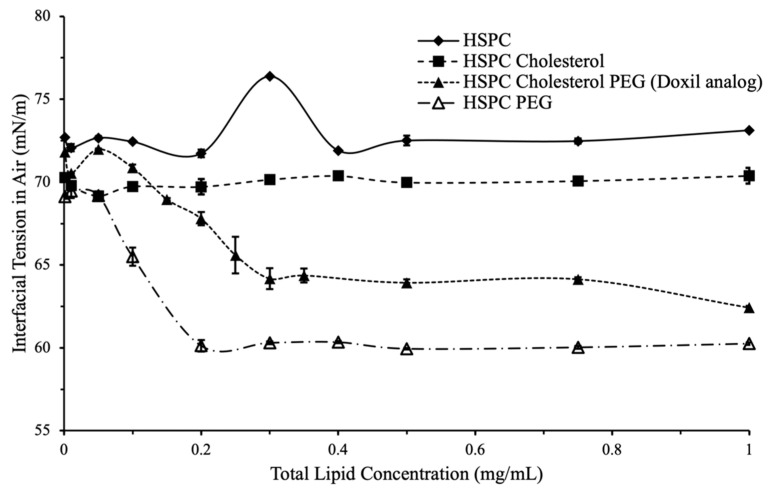
Interfacial tension vs. total lipid concentration for variations in formulation components. (closed diamond) No effect on surface tension was observed for HSPC only, or HSPC cholesterol liposomes. (closed square) A surfactant-like effect was observed for liposomes with PEG (open triangle). Liposomes with both PEG and cholesterol (Doxil^®^ analog, closed triangle) had a lower overall interfacial tension compared to other formulations.

Doxil^®^ analog formulations exhibited surfactant-like behavior with interfacial tension decreasing with increasing liposome concentration following a sigmoidal profile (Figure 2). Surfactant-type behavior as observed here was expected due to the small size of liposomes and high surface energy; however, this behavior was not observed in formulations that contained little or no PEG.

To confirm this, 80, 100, and 120 nm non-PEGylated silica nanoparticles suspended in HPLC-grade water were used and interfacial tension was measured using the same method as the liposomes. The data confirmed that non-PEGylated nanoparticles do not demonstrate a concentration-dependent effect on interfacial tension that is characteristic of surfactants and surface-active particles.

### 2.3. Effect of PEG and Cholesterol on Interfacial Tension

**Figure 3:**
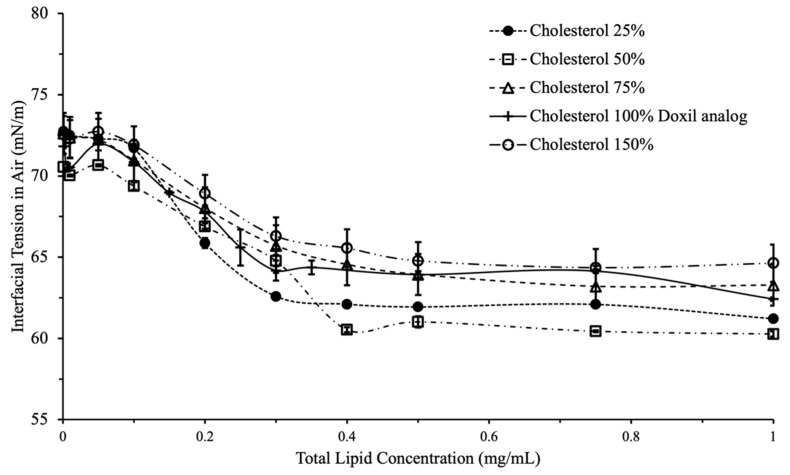
Interfacial tension vs. total lipid concentration for variations in cholesterol content. Overall decrease in IFT did not differ significantly with increases in cholesterol.

Variation in PEG content, 25–150% of PEG content in Doxil^®^, was used to study the effect of PEG on liposomal interfacial tension at different liposomal concentrations (Figure 3). At 25% PEG content, a non-significant decrease in overall interfacial tension with total lipid concentration (71.5 mN/m at 0.001mg/mL lipid to 71.6 mN/m at 1mg/mL) was observed, compared to liposomes with PEG content ≥50% (Figure 3).

PEG was found to play an important role in liposomal interfacial tension. Changes in interfacial tension begin to appear in liposomes with PEG content 25–50% compared to Doxil^®^. This provides a range where PEG starts contributing towards the interfacial activity of liposomes; the exact concentration would likely differ with liposomes encapsulating different drugs.

Based on the data shown here, interfacial tension is a valuable tool to assess the effect of PEG content on surface activity of liposomes. Since the reason for adding PEG in liposomal formulations is to prevent protein adsorption and prolong circulation, it is likely that interfacial tension can be a quality assurance tool to quantify this effect.

**Figure 4:**
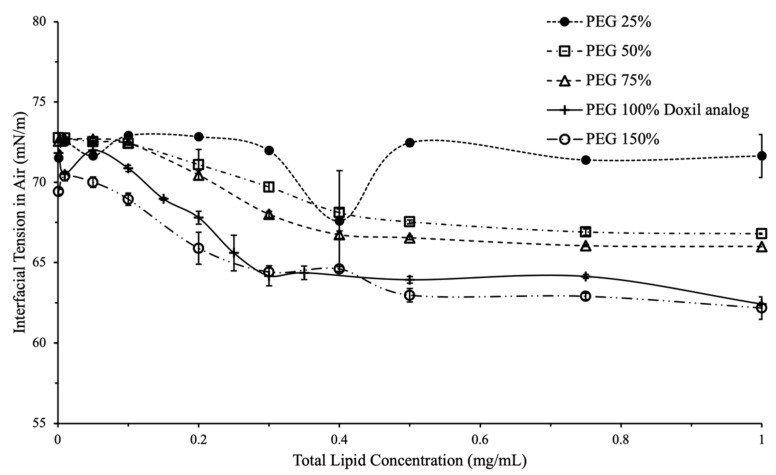
Interfacial tension vs. total lipid concentration for variations in PEG content. A larger decrease was seen in IFT for liposomes that had 50% or greater PEG content of Doxil^®^ analog.

Cholesterol had a less pronounced effect on liposomal interfacial tension compared to PEG. Presence of cholesterol in the liposomes led to a small reduction in interfacial tension. (Figure 4). No concentration-dependent effect was observed, which indicates the presence of cholesterol neither reduces nor increase the surface activity of liposomes. Accordingly, interfacial tension is likely not an appropriate tool to assess the efficacy of cholesterol incorporation in liposomes.

### 2.4. Polar and Nonpolar Contributions to Interfacial Tension

**Figure 5:**
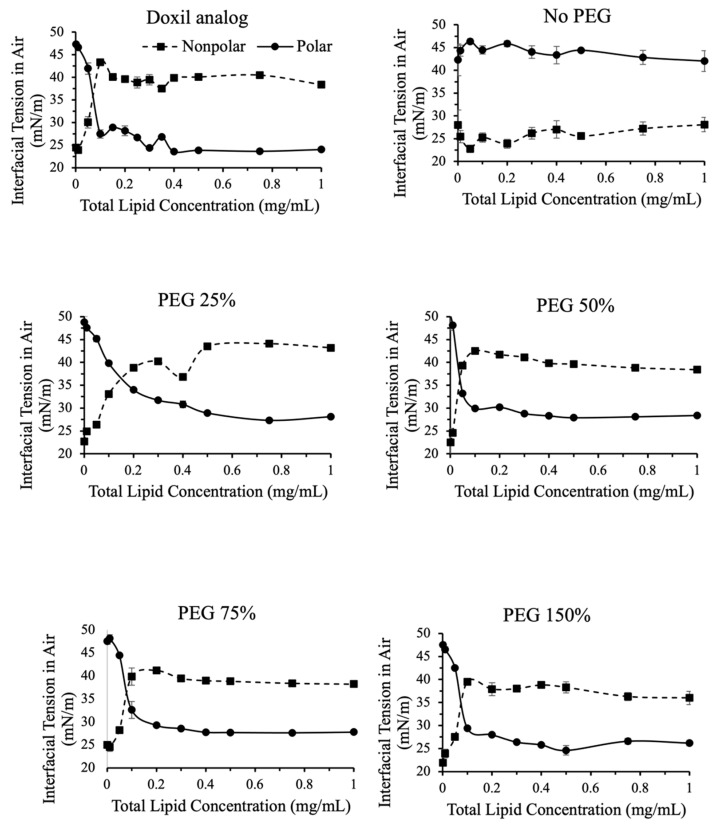
Polar and nonpolar contributions toward interfacial tension for liposomes with variations in PEG content. Contributions were calculated using IFT measurements in air and in dodecane. Nonpolar contributions toward IFT increased in PEG containing formulations greater than 0.1 mg/mL. Nonpolar contributions were greater than polar contributions in all PEG containing formulations for concentrations greater than 0.2 mg/mL.

**Figure 6:**
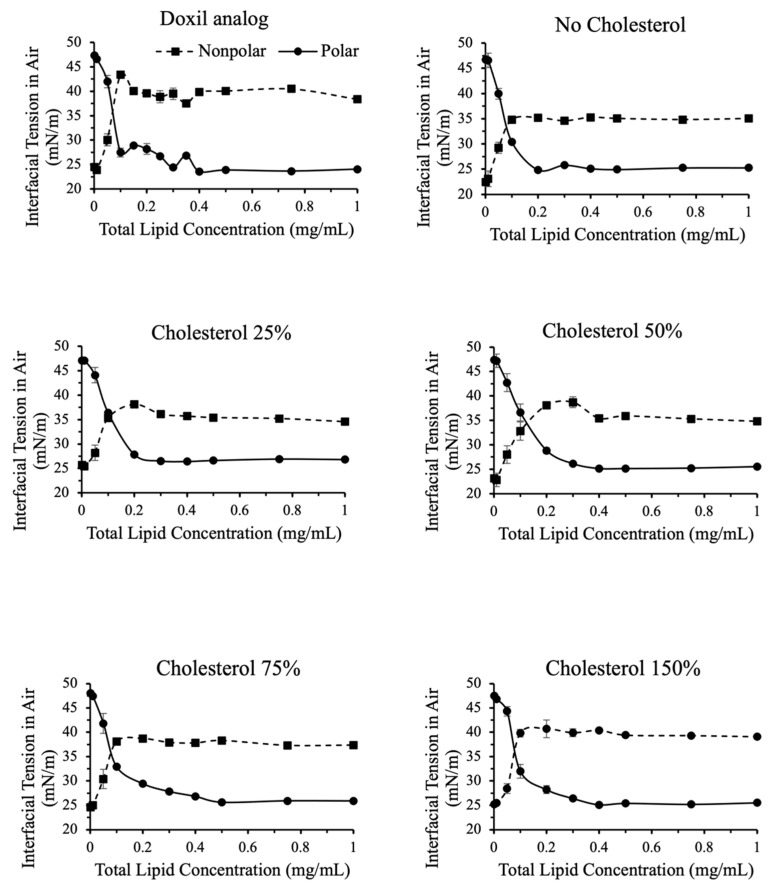
Polar and nonpolar contributions toward interfacial tension for liposomes with variations in cholesterol content.

Figure 5 and Figure 6 show a breakdown of polar and nonpolar contributions to interfacial tension for liposomal formulations with varying PEG and cholesterol contents, respectively.

In liposomes that contained PEG, the non-polar component of interfacial tension increased with increasing concentration (Figure 5). The contribution of the non-polar component of interfacial tension increased between total lipid concentrations 0.01–0.2 mg/mL, and then plateaued. The reverse was true for the polar component of interfacial tension. This indicates that as the surface saturates with liposomes it becomes less polar, as expected. The amount of PEG did not affect the breakdown of polar and nonpolar contributions to interfacial tension.

In the absence of PEG, no change in polar and nonpolar contributions to interfacial tension was observed with increasing liposome concentrations.

As shown in Figure 6, similar observations were made when varying cholesterol content. For cholesterol more than 50% compared to Doxil^®^, a slight increase in nonpolar contribution to interfacial tension was observed. This indicates that, unlike total interfacial tension, nonpolar contribution to surface tension may be a good valuable quality assurance tool to assess the effect of cholesterol content.

## 3. Materials and Methods

Liposomal doxorubicin (Cat No. PHP020DX), a research-grade equivalent of Doxil^®^, was purchased from Liposomics, ProFoldin (Hudson, MA, USA). The reported hydrodynamic diameter and polydispersity index (PDI) of the liposomal doxorubicin were 92 nm and 0.114, respectively. HSPC (Coatsome^®^ NC-21E) and PEG (Sunbright^®^ DSPE-020CN) were purchased from NOF Corporation (Tokyo, Japan). Cholesterol (Cat No. 101382) was purchased from MP Biomedicals, LLC (Irvine, CA, USA). Chloroform (HPLC Grade) was purchased from Fisher Chemical (Fair Lawn, NJ, USA). Phosphate-buffered saline (PBS) 10X, Fisher Bioreagents, was diluted to 1X using HPLC Grade Water (Catalog No. W5-4, Fisher Chemical), and adjusted to a final pH between 7.3–7.4. n-Dodecane (Catalog No. 117592500) was purchased from Acros Organics (Verona, Italy).

### 3.1. Formulation and Size of Liposomes

Doxil^®^ (HSPC, cholesterol, PEG 9.6:3.2:3.2 weight ratio) was used as a reference liposomal formulation. In-house liposome formulations (Table 1) were designed to resemble the composition of Doxil^®^ with slight variations in PEG content, cholesterol content, and size. All in-house liposome formulations were made using the thin-film hydration method. Rotary evaporator R-300, Buchi Switzerland, was used to make lipid films. Clear stock solutions for each lipid, HSPC, cholesterol, and DSPE-PEG, of concentration 20 mg/mL in chloroform, were mixed in a 100 mL round bottom flask. Volume of each lipid stock solution was determined by calculating the amount of lipid in 3 mL of final formulation with concentrations mentioned in Table 1. For example, for all formulations, 1.44 mL of HSPC-chloroform (28.8 mg HSPC) stock solution was used to achieve the desired concentration of HSPC 9.6 mg/mL in 3 mL MLVs. The organic solvent was evaporated using rotary evaporation, with water bath at 60 °C, above the gel-liquid crystal transition temperature (T_m_) of the lipid, such that a uniform thin lipid film was formed on the sides of the flask. To make sure that no residual organic solvent was left, we allowed the film to dry further under vacuum for 45 min, followed by drying using nitrogen. MLVs were then obtained by hydrating the dry lipid film with PBS 1X, pH 7.3, using a vortex. The MLVs were then stored at 4 °C to be later downsized using extrusion. Whatman membranes, 400 nm (Catalog No. 800282), 200 nm (Catalog No. 800281), 100 nm (Catalog No. 800309), and 50 nm (Catalog No. 800308) were in a mini-extruder setup by Avanti Polar Lipids, Inc. (Catalog No. 610000) for downsizing the MLVs to approximately 100 nm and 200 nm with a uniform PDI (0.02–0.04), Table 1.

### 3.2. Size Characterization of Liposomes

Dynamic light scattering (DLS) was used to characterize the nanoparticles for average size (hydrodynamic diameter) and size distribution (polydispersity index, PDI). A Mobius-122 particle size analyzer by Wyatt Technology was used. The instrument is equipped with a 532 nm laser light source and a 163.5° maximum measurement angle. A 75 uL sample was placed in a quartz cuvette and analyzed. Dynamics software (Version 7.8.2.18) by Wyatt Technology was used to operate the instrument and obtain intensity-particle size distribution (ISD) profiles. Each DLS measurement consisted of 100 scans each of 1 s acquisitions; five measurements were used to obtain mean hydrodynamic diameter and mean PDI for each sample.

### 3.3. Interfacial Properties of Liposomes

Liposomal interfacial properties were quantified using the pendant drop method. The instrument utilized in this study, the OCA25 by DataPhysics, recorded a 25 µL drop over a period of 70 s at 8.7 frames per second. All measurements were obtained at ambient temperature. The “optical trigger start” setting was used to record the video; the video recorded automatically for 70 s as soon as the drop reached the trigger line set on screen. Before each formulation was measured, IFT measurements of HPLC-grade water was used for calibration and IFT values between 70–72 mN/m were accepted. Each formulation was diluted to a total lipid concentration of 0.001–1 mg/mL and the IFT in air and in dodecane was measured in triplicate at each concentration. A glass cube filled with dodecane was used to completely immerse the needle before dispensing the sample drop.

### 3.4. Data Analysis

Time 0 in the video was set as soon as 25 µL of sample was completely dispensed. Software SCA, (Version 5.0.41.5041) by DataPhysics was used to process the recorded videos for each drop from time 0 and IFT values in air and dodecane were obtained. Dispersive contributions to IFT were calculated from the measured IFT values in air and in dodecane using Good’s equation for two-component interfacial tension. Polar contributions to interfacial tension were calculated by subtracting the dispersive contribution (obtained from Equation (1)) from interfacial tension of sample (Equation (2)).

Equation (1)—Good’s equation for two-component interfacial tension:

γ_1, 2_ = γ_1_ + γ_2_ − 2[(γ_1_^D^ γ_2_^D^)^1/2^ + (γ_1_^P^ γ_2_^P^)^1/2^], (1)

where, γ_1_ = measured interfacial tension of sample in air,

γ_2_ = surface tension of n-dodecane,
γ_1,2_ = measured interfacial tension of sample in dodecane,
γ_1_^D^ γ_1_^P^ = dispersive and polar component of sample,
γ_2_^D^ γ_2_^P^ = dispersive and polar component of dodecane,
γ_1,2_ = γ_1_ + γ_2_ − 2[(γ_1_^D^ γ_2_^D^)^1/2^], since γ_2_^P^ = 0.

Equation (2)—polar contribution to interfacial tension:

γ_1_^P^ = γ_1_ − γ_1_^D^. (2)

Raw data obtained from SCA25 consisted of IFT values at time points 0–70 s. A scatter plot was used to obtain a raw data curve for each measurement by plotting IFT values against time. All IFT time curves exhibited a similar profile of rapid IFT decay followed by a plateau at the 40–50 s mark. To obtain a representative IFT value from the raw data, we calculated the average IFT value over a 10 s duration after the IFT curve plateaued. Mean IFT values obtained from all three measurements along with standard error were calculated for all samples in air and in dodecane and plotted against different concentrations (0.001–1 mg/mL) for that formulation.

Interfacial tension was also plotted against total lipid concentration; a sigmoidal curve was observed for most samples. Two additional calculations were performed from the interfacial tension-lipid concentration data: the average interfacial tension after plateau, and the lipid concentration at which the polar and non-polar contributions to interfacial tension were equal. Each sample was measured in triplicate with mean and standard error reported.

## 4. Conclusions

The main conclusion was that PEGylation has a major effect on interfacial tension. Accordingly, interfacial tension can be used to assess the effectiveness of PEGylation in terms of surface activity. Furthermore, the surfactant-like behavior of liposomes is lost in non-PEGylated formulations. In the case of cholesterol, the effect was less pronounced and only observed when looking at the nonpolar contribution to interfacial tension rather than total interfacial tension. Through varying formulation concentration, composition, and particle size, we identified four interfacial tension-related measures that may potentially be used for physicochemical characterization:

1. Absolute interfacial tension value at plateau;
2. Rate of interfacial tension decay after droplet formation;
3. Non-polar and polar contributions to interfacial tension;
4. Liposome concentration at which polar and nonpolar contributions are equal.

To our knowledge, this work investigates for the first-time interfacial tension as a quality assurance tool for liposomal formulations and may serve as a baseline for additional investigations to expand this concept to other nanoparticle-based drug products.

## Figures and Tables

**Table 1:** Lipid composition, mean hydrodynamic diameter (Hd), and PDI. Doxil^®^ composition (HSPC, cholesterol, PEG as 9.6/3.2/3.2 mg/mL) was used as reference formulation.

Formulation	HSPC (mg/mL)	Cholesterol (mg/mL)	PEG (mg/mL)	Mean H_d_ (nm) ± SE	Mean PDI ± SE
Doxil^®^ analog	9.6	3.2	3.2	107.7 ± 0.3	0.04 ± 0.0
HSPC only	9.6	0	0	86.6 ± 0.75	0.03 ± 0.002
HSPC Chol	9.6	3.2	0	102.4 ± 0.45	0.04 ± 0.0
PEG 25% of Doxil^®^	9.6	3.2	0.8	99.4 ± 0.3	0.02 ± 0.005
PEG 50% of Doxil^®^	9.6	3.2	1.6	88.8 ± 0.3	0.04 ± 0.002
PEG 75% of Doxil^®^	9.6	3.2	2.4	93.7 ± 0.7	0.04 ± 0.003
PEG 150% of Doxil ^®^	9.6	3.2	4.8	100.4 ± 1.11	0.032 ± 0.004
HSPC PEG	9.6	0	3.2	104.9 ± 0.6	0.032 ± 0.01
Cholesterol 25% of Doxil^®^	9.6	0.8	3.2	102.9 ± 0.2	0.04 ± 0.0
Cholesterol 50% of Doxil^®^	9.6	1.6	3.2	105.3 ± 0.19	0.032 ± 0.01
Cholesterol 75% of Doxil^®^	9.6	2.4	3.2	92.4 ± 0.4	0.03 ± 0.004
Cholesterol 150% of Doxil^®^	9.6	4.8	3.2	96.2 ± 0.6	0.03 ± 0.004
Doxil^®^ analog, larger size	9.6	3.2	3.2	201.7 ± 1.8	0.03 ± 0.004
Liposomal doxorubicin *	9.6	3.2	3.2	92	0.114

* Liposomal doxorubicin data per Profoldin.

**Table 2:** Slope of interfacial tension for the first ten seconds.

	Drop 1	Drop 2	Drop 3	Average	SE
Doxil^®^ analog	−0.358	−0.327	−0.357	−0.347	0.013
Doxil^®^ analog 200 nm	−0.229	−0.271	−0.262	−0.254	0.016
Liposomal doxorubicin	−0.089	−0.087	−0.112	−0.096	0.010

## Data Availability

Data is contained within the article.
